# Exogenous Calcium Enhances Castor Tolerance to Saline–Alkaline Stress by Regulating Antioxidant Enzyme Activity and Activating Ca^2+^ and ROS Signaling Crosstalk

**DOI:** 10.3390/ijms252312717

**Published:** 2024-11-26

**Authors:** Fei Hao, Zhigang Cui, Xuan Dong, Yan Gao, Rongjin Wang, Hui Zhang, Guolin Lin

**Affiliations:** 1 College of Land and Environment, Shenyang Agricultural University, Shenyang 110866, China; haofei@stu.syau.edu.cn (F.H.); 2022200137@stu.syau.edu.cn (Z.C.); 2019200115@stu.syau.edu.cn (X.D.); gaoyan2915@stu.syau.edu.cn (Y.G.); 2021220451@stu.syau.edu.cn (R.W.); huizhang@syau.edu.cn (H.Z.); 2 Panxi Crops Research and Utilization Key Laboratory of Sichuan Province, Xichang University of Sichuan Province, Xichang 615000, China

**Keywords:** castor, saline–alkaline stress, Ca^2+^ signaling, ROS signaling, APX3, CAT2

## Abstract

Saline–alkaline stress is a major factor limiting agricultural development, with calcium (Ca^2+^) playing a role in regulating plant tolerance through multiple signaling pathways. However, the specific mechanisms by which Ca^2+^ mediates saline–alkaline stress tolerance at the molecular level remain incompletely understood. This study investigates the effects of exogenous Ca^2+^ application on enhancing plant tolerance to saline–alkaline stress, focusing on its impact on the antioxidant system and Ca^2+^ and reactive oxygen species (ROS) signaling pathways. Through physiological assays and transcriptomic analyses, we evaluated oxidative damage markers, antioxidant enzyme activities, and the expression of key Ca^2+^ and ROS signaling genes. The results showed that saline–alkaline stress significantly elevated ROS levels, which led to increased membrane lipid peroxidation and induced upregulation of antioxidant response elements in castor roots. Exogenous calcium treatment reduced ROS accumulation by increasing superoxide dismutase (SOD), peroxidase (POD), and catalase (CAT) activities and decreasing malondialdehyde (MDA) levels, demonstrating a marked improvement in the antioxidant system. Transcriptomic analysis identified *CAT2* (LOC107261240) as the primary target gene associated with increased CAT activity in response to exogenous calcium. Additionally, the upregulation of specific Ca^2+^ channels, Ca^2+^ sensors, ROS receptors, and antioxidant-related genes with calcium treatment highlights the critical role of Ca^2+^–ROS signaling crosstalk in enhancing stress tolerance. Protein–protein interaction analysis identified *APX3* and other hub genes involved in Ca^2+^–ROS signaling transduction and the regulation of antioxidant activity. These findings enhance our understanding of calcium’s complex regulatory roles in plant abiotic stress responses, offering new theoretical insights for improving crop resilience in agriculture.

## 1. Introduction

Saline–alkaline soil has a detrimental effect on plant growth and crop yield. With the growing impact of climate change, soil salinization has emerged as a global ecological problem [1]. Sodium bicarbonate (NaHCO_3_), present in soil, is commonly recognized as a mixed stress factor for plants [2]. Alkaline salts, compared to neutral salts, can present a greater threat to plant growth [3,4]. Salt stress impacts are seen in ion toxicity and osmotic stress. Besides the ion toxicity and osmotic stress induced by neutral salts, alkaline salts also cause pH and HCO^3−^/CO_3_^2−^ stress, exacerbating secondary oxidative damage in plants [5,6]. Research on saline–alkaline stress in plants has predominantly focused on neutral salts, with limited studies on alkaline salts. Bioremediation of saline–alkaline soil is viewed as a potential solution [7,8]. Plants have evolved multiple antioxidant systems to combat the excessive accumulation of reactive oxygen species (ROS) under abiotic stress. Furthermore, plants have developed various signaling networks to initiate adaptive responses under stress [9]. However, plants’ natural resistance is inherently limited, and elevated saline–alkaline stress concentrations severely inhibit growth. Applying exogenous substances is a cost-effective, efficient, and convenient approach, which has been widely adopted in agriculture to improve plant tolerance [10].

Calcium (Ca^2+^) serves as both an essential nutrient and a crucial signaling molecule in plants, participating in a wide range of metabolic and stress response pathways. Extensive research has demonstrated that calcium improves antioxidant enzyme activity and mitigates oxidative damage caused by abiotic stresses [11,12,13]. In addition, calcium can function as a signal, engaging in crosstalk with other signals to alleviate salt stress-induced oxidative damage [14]. Cytosolic Ca^2+^ transients are primarily regulated by calcium-permeable channels located on the plasma membrane and internal organelles [15]. Various Ca^2+^ permeable channel families are positioned on the PM, supporting Ca^2+^ influx from the apoplast and possibly residing on internal membranes like the ER, GA, mitochondria, chloroplasts, vacuoles, and peroxisomes. Research suggests that Ca^2+^ root pretreatment may trigger Ca^2+^ signaling by allowing external Ca^2+^ to enter the cytosol [16]. Calcium signals are detected by calcium sensors, including Ca^2+^-dependent protein kinases (CDPKs), calmodulin (CaM), and calcineurin B-like (CBL) proteins, which convert them into downstream defense responses [17]. Calcium sensors serve as key nodes in plant stress responses [18]. For example, salt-induced oxidative stress is mediated by Ca^2+^/calmodulin (CaM) signaling pathways [19]. The overexpression of CPK and CML enhances plant stress tolerance, reflected in the removal of excessive ROS [20,21]. Currently, studies on exogenous calcium’s role in enhancing plant tolerance to saline–alkaline stress focus largely on physiology, with limited research at the transcriptional level.

An excess of ROS is harmful to plants; however, when kept at lower levels, ROS acts as a signaling molecule, regulating plant growth, development, and adaptation to unfavorable conditions [22]. ROS signaling pathways are categorized as external (apoplast and cell wall), internal (cytoplasm and nucleus), and organelle-based (chloroplasts, mitochondria, peroxisomes, and other organelles) [23]. External stress activates ROS production in the initial cell via respiratory burst oxidase homologs (RBOHs) and facilitates the diffusion of H_2_O_2_ produced in the apoplast into cells through H_2_O_2_-INDUCED CA^2+^ INCREASES 1 (HPCA1) and Aquaporins (AQPs). Intracellular H_2_O_2_ and Ca^2+^ signals interact within each cell, prompting further ROS generation [24]. Research has found that exogenous melatonin and spermidine can enhance plant stress tolerance via RBOHF- and RBOH1-mediated ROS signaling [25,26]. Furthermore, the ability of ROS signaling to interact with extracellular calcium concentration-induced Ca^2+^ signaling in response to abiotic stress has been well reviewed [27,28]. ROS receptor proteins sense reactive oxygen species through distinct signaling pathways and transmit this information to regulate downstream redox responses. Recently, the identification of the ROS receptor HPCA1 has brought attention to the crosstalk between ROS and Ca^2+^ signaling in enhancing plant stress tolerance [29]. However, the role of exogenous calcium in regulating Ca^2+^ and ROS signaling under saline–alkaline stress is not yet understood.

Castor (*Ricinus communis* L.) is an industrial crop and a pioneer plant for the reclamation of saline–alkaline soil. The castor plant’s root system is extensive, grows quickly, and shows a high tolerance to abiotic stress. Consequently, castor should be grown in marginal areas to increase land utilization and improve soil. In this context, tolerance to abiotic stress is especially crucial [30]. The seedling stage is the most sensitive period for castor under saline–alkaline stress [31]. Thus, improving tolerance to saline–alkaline stress during the seedling stage is vital for castor’s growth and stress resistance across the growth cycle.

In this study, physiological and transcriptomic methods were employed to examine how exogenous calcium affects antioxidant enzymes in castor roots under saline–alkaline stress and regulates Ca^2+^ and ROS signaling. The study hypothesizes the following: (1) that exogenous calcium enhances antioxidant enzyme activity, alleviating secondary oxidative stress caused by saline–alkaline stress; and (2) that exogenous calcium activates Ca^2+^ and ROS signaling, contributing to castor’s tolerance to saline–alkaline stress. To test these hypotheses, we analyzed oxidative damage markers, antioxidant enzyme activities, and gene expression associated with Ca^2+^ and ROS signaling.

## 2. Results

### 2.1. Effect of Exogenous Calcium on the Antioxidant System of Castor Root Under Saline-Alkaline Stress

To investigate the impact of exogenous calcium on the antioxidant system of plants in response to saline–alkaline stress. The activities of three antioxidant enzymes, superoxide dismutase (SOD), peroxidase (POD), and catalase (CAT), as well as the malondialdehyde (MDA), hydrogen peroxide (H_2_O_2_), and superoxide anion (O_2_^−^) contents, were assessed in response to various interventions.

**Figure 1:**
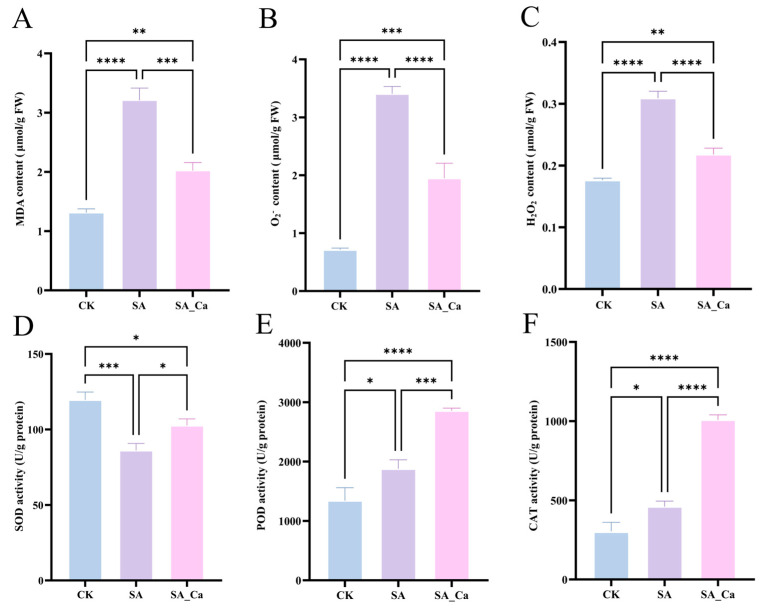
Effects of Ca^2+^ on the antioxidant system of castor roots under saline–alkaline stress. (**A**) malondialdehyde (MDA) content; (**B**) superoxide anion (O_2_^−^) content; (**C**) hydrogen peroxide (H_2_O_2_) content; (**D**) superoxide dismutase (SOD) activity; (**E**) peroxidase (POD) activity; (**F**) catalase (CAT) activity. CK: control; SA: saline–alkaline stress; SA_Ca: saline–alkaline stress with CaCl_2_ treatment. Each treatment contains three biological repeats. The one-way ANOVA and Tukey’s new multiple-range method were used to compare the differences between treatments. Data are presented as mean ± SE (* *p*  <  0.05, ** *p*  <  0.01, *** *p * <  0.001, **** *p*  <  0.0001).

Saline–alkaline stress significantly increased the contents of MDA, O_2_^−^, and H_2_O_2_ in castor roots (*p* < 0.0001) (Figure 1A–C). Additionally, it significantly enhanced the activities of POD and CAT in the root system (*p* < 0.05). However, SOD activity was significantly reduced under saline–alkaline stress (*p* < 0.001) (Figure 1D–F). This suggested that the plant’s antioxidant enzyme activity was bolstered to withstand the external saline–alkaline hazards, and the degree of cell membrane lipid peroxidation was intensified as a result of the excessive accumulation of ROS in castor under saline–alkaline stress. The exogenous calcium treatment significantly reduced the MDA (*p* < 0.001), O_2_^−^, and H_2_O_2_ content (*p* < 0.0001) in castor seedlings. When compared to SA therapy, the levels of MDA, O_2_^−^, and H_2_O_2_ decreased by 36.99%, 42.76%, and 29.44%, respectively. The activity of SOD, POD, and CAT increased by 19.16%, 52.06%, and 121.05%, respectively. Nonetheless, the SA_Ca treatment’s SOD activity did not return to the CK level. The results suggested that exogenous calcium primarily enhanced POD and CAT activities, scavenged accumulated ROS, and decreased MDA content in castor seedlings under saline–alkaline stress.

### 2.2. Transcriptome Sequencing and Differential Expression of Genes (DEGs) Identification

**Figure 2:**
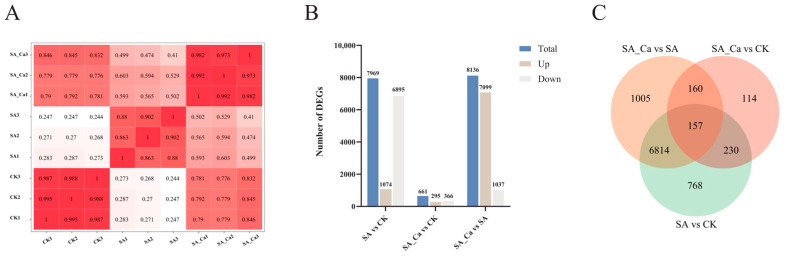
Transcriptome data analysis and screening of differentially expressed genes (DEGs). (**A**) Pearson’s correlation coefficient analysis. (**B**) Bar graph of up- and down-regulated DEGs from pairwise comparison groups. (**C**) Venn graph for up- and down-regulated DEGs from the pairwise comparison groups.

To further clarify the possible mechanism of calcium under saline–alkaline stress in castor plants, transcriptome sequencing was performed on castor seedling roots. Root samples were collected for RNA sequencing after 36 h of each treatment, totaling nine samples. Across the nine sequencing libraries, raw read counts ranged from 37,899,606 to 51,277,888, with bases of quality value ≥ 30 (Q30%) accounting for over 95% and GC content exceeding 43% (Table S1). Following read quantification, Pearson correlation analysis of the three replicates demonstrated high sequencing data quality, suitable for further analysis (Figure 2A). DEGs of three two-by-two comparison groups (SA vs. CK, SA_Ca vs. CK, and SA_Ca vs. SA) were obtained based on the screening conditions of |log2FC| > 2 and *p* < 0.05. The distributions of differentially expressed genes among distinct treatment groups are illustrated in Figure 2B. The SA vs. CK, SA_Ca vs. CK, and SA_Ca vs. SA comparisons revealed 7969 (1074 upregulated, 6895 downregulated), 661 (295 upregulated, 366 downregulated), and 8136 (7099 upregulated, 1037 downregulated) DEGs, respectively. A Venn diagram was created based on the DEGs from the three comparison groups (Figure 2C). In the SA vs. CK and SA_Ca vs. SA groups, 6971 shared DEGs were identified, considered as DEGs affected by saline–alkaline stress, and regulated by exogenous calcium under stress, providing a basis for further analysis.

### 2.3. Gene Ontology (GO) and Kyoto Encyclopedia of Genes and Genomes (KEGG) Enrichment Analysis of DEGs

**Figure 3:**
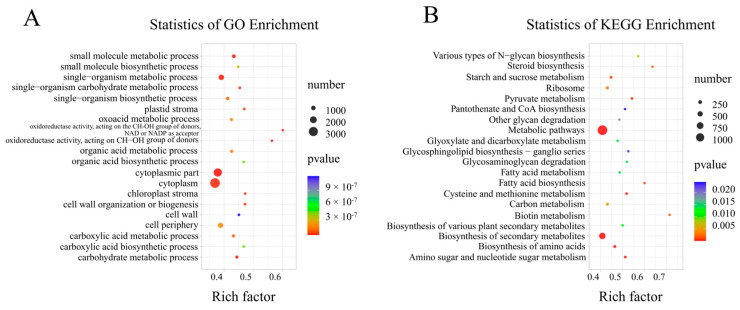
Two paired comparison groups shared GO and KEGG enrichment analysis of DEGs. The horizontal axis indicates the degree of enrichment (enrichment factor); the vertical axis indicates the enriched GO and KEGG pathways; the size of the dots indicates the number of differentially expressed genes enriched in the pathways; and the color of the dots indicates different *p* values. (**A**) GO enrichment analysis of DEGs. (**B**) KEGG pathway enrichment of DEGs. Two pairwise comparison groups: SA vs. CK and SA_Ca vs. SA (experimental group vs. control group).

GO and KEGG enrichment analyses were conducted on the DEGs. The DEGs were significantly enriched in 56 GO terms, with 24 BP terms, 14 MF terms, and 17 CC terms (*p* < 0.05) (Figure S1). The top 20 enriched GO terms showed that DEGs were primarily enriched in small molecule metabolic processes (GO:0044281), the cytoplasmic part (GO:0044444), and oxidoreductase activity acting on CH-OH groups with NAD or NADP as acceptor (GO:0016616) (Figure 3A). A total of 28 significantly enriched KEGG pathways were identified (*p* < 0.05) (Table S2). The top 20 KEGG-enriched pathways indicated that DEGs were primarily enriched in metabolic pathways (ko01100), biosynthesis of secondary metabolites (ko01110), and amino sugar and nucleotide sugar metabolism (ko00520) (Figure 3B).

### 2.4. DEGs Associated with ROS Metabolism Under Different Treatments

**Figure 4:**
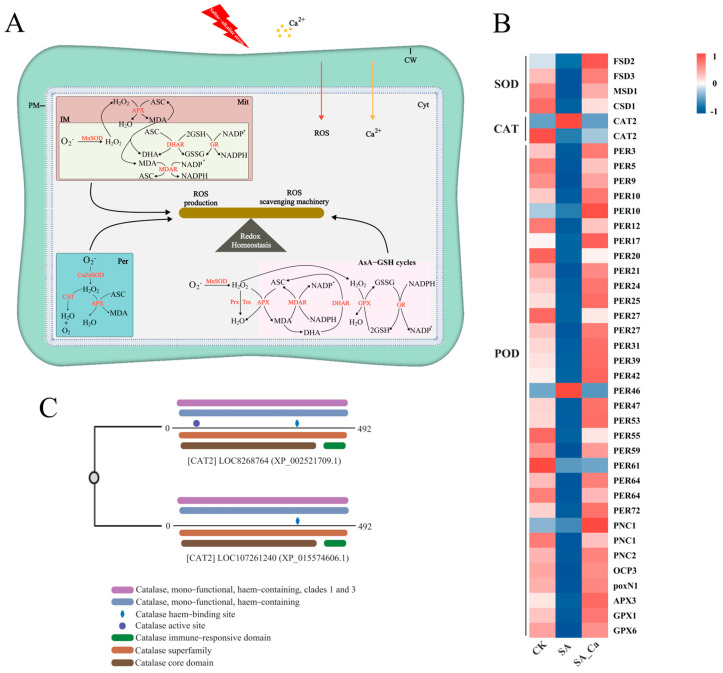
Regulation of ROS metabolism by exogenous calcium. (**A**) Ca^2+^ signaling and ROS signaling synergistically regulate intracellular redox homeostasis. (**B**) DEGs associated with SOD, CAT, and POD. (**C**) Amino acid sequence structural domain analysis of two CAT2 genes. CW, cell wall; Mit, mitochondrion; IM, inner mitochondrial membrane; Cyt, cytoplasm; Per, peroxisome; APX, ascorbate peroxidase; ASC, ascorbate; DHA, dehydroascorbate; DHAR, dehydroascorbate reductase; NADPH, nicotinamide adenine dinucleotide phosphate; GPX, glutathione peroxidase; GSH, reduced glutathione; GSSG, oxidized glutathione; GR, glutathione reductase; Prx, peroxiredoxin; Trx, thioredoxin reductase.

To clarify the mechanism behind calcium-induced antioxidant enzyme activity, we analyzed ROS scavenging-related genes in the transcriptome data. Saline–alkaline stress suppressed the expression of most antioxidant enzyme genes, but calcium pretreatment increased their expression (Figure 4B). This included four SOD genes, two CAT genes, and 33 POD genes. Interestingly, the two detected CAT genes exhibited opposite expression trends, and among the 33 detected POD genes, *PER46* showed an expression trend opposite to the others. Additionally, genes associated with other antioxidant enzymes (MDAR, DHAR, GR, GST, PRX, TRX) were also upregulated under calcium treatment (Figure S2).

To investigate the distinct roles of the two CAT2 genes, we obtained the amino acid sequences of the differentially expressed *CAT2* (LOC8268764) and *CAT2* (LOC107261240) genes from the castor reference protein sequence and then performed multiple sequence alignment on these two genes (Figure S3). A domain analysis was subsequently performed on them (Figure 4C). It was found that the CAT active site domain is unique to the *CAT2* (LOC8268764) gene. The correspondence between LOC ID and Gene Name is shown in Supplementary Table S3.

### 2.5. DEGs Associated with Signal Transduction Under Different Treatments

Ca^2+^ and ROS signaling are interrelated in controlling plant resistance to environmental stress. To clarify the interaction between Ca^2+^ and ROS signaling under saline–alkaline stress, we analyzed transcriptome sequencing results for genes related to ROS and Ca^2+^ signaling.

**Figure 5:**
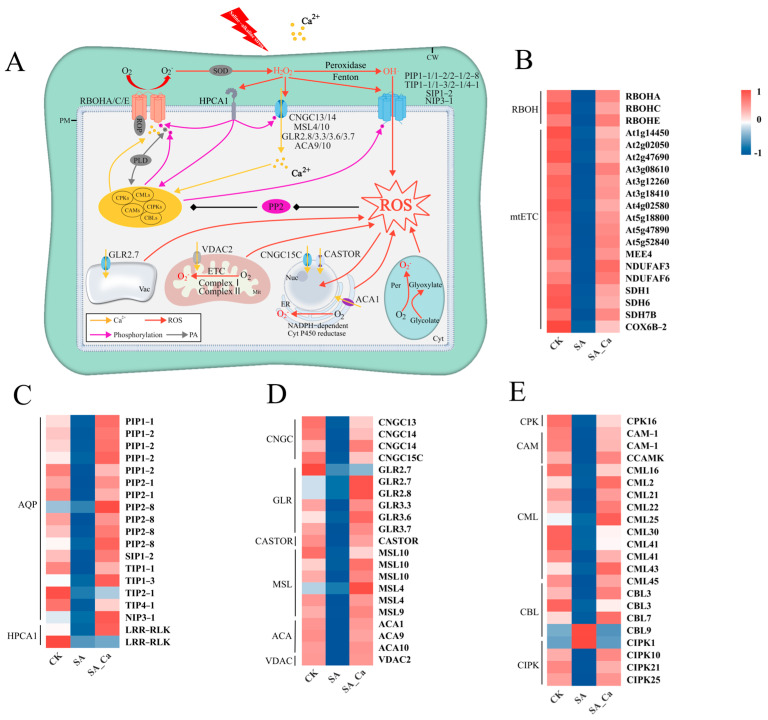
Regulation of intracellular signaling by exogenous calcium. (**A**) Intracellular Ca^2+^ signaling crosstalk with ROS signaling. (**B**) DEGs associated with ROS generation. (**C**) DEGs associated with ROS sensors. (**D**) DEGs associated with calcium ion channels. (**E**) DEGs associated with Ca^2+^ signaling sensors. RBOH, respiratory burst oxidase homolog; mtETC, mitochondrial electron transport chain; PIP, plasma membrane intrinsic protein; TIP, tonoplast intrinsic protein; SIP, small basic intrinsic protein; NIP, NOD26-like intrinsic protein; CNGC, cyclic nucleotide-gated channel; GLR, glutamate receptor-like protein; MSL, mechanosensitive channel; ACA, Ca^2+^ ATPases; VDAC, voltage-dependent anion channel; CASTOR, plant nuclear ion channel.

Our study detected 21 genes related to ROS generation (Figure 5B), including three genes (RBOH) involved in ROS production in the apoplast and 17 genes (mtETC) related to mitochondrial ROS generation. Furthermore, 19 genes related to ROS sensors were identified (Figure 5C), comprising 17 ROS channel genes (AQP) and two ROS receptor genes (HPCA1), all of which were downregulated in the SA treatment and upregulated in the SA_Ca treatment.

Relative to the SA treatment, the SA_Ca treatment led to an upregulation in calcium ion channel-related gene expression on cell and organelle membranes, including four cyclic nucleotide-gated channels (CNGC), six glutamate receptor-like proteins (GLR), six mechanosensitive channels (MSL), three Ca^2+^ ATPases (ACA), one voltage-dependent anion channel (VDAC), and one plant nuclear ion channel (CASTOR) (Figure 5D). Ca^2+^ sensors are crucial in the crosstalk between Ca^2+^ and ROS signaling. In our study, 21 Ca^2+^ sensor-related genes were detected, with all genes except *CBL9* and *CIPK1* downregulated in the SA group and upregulated in the SA_Ca group (Figure 5E). Furthermore, most signaling hub genes were suppressed by saline–alkaline stress and upregulated under exogenous calcium treatment (Figure S4).

### 2.6. Protein-Protein Interaction Network Analysis (PPI)

**Figure 6:**
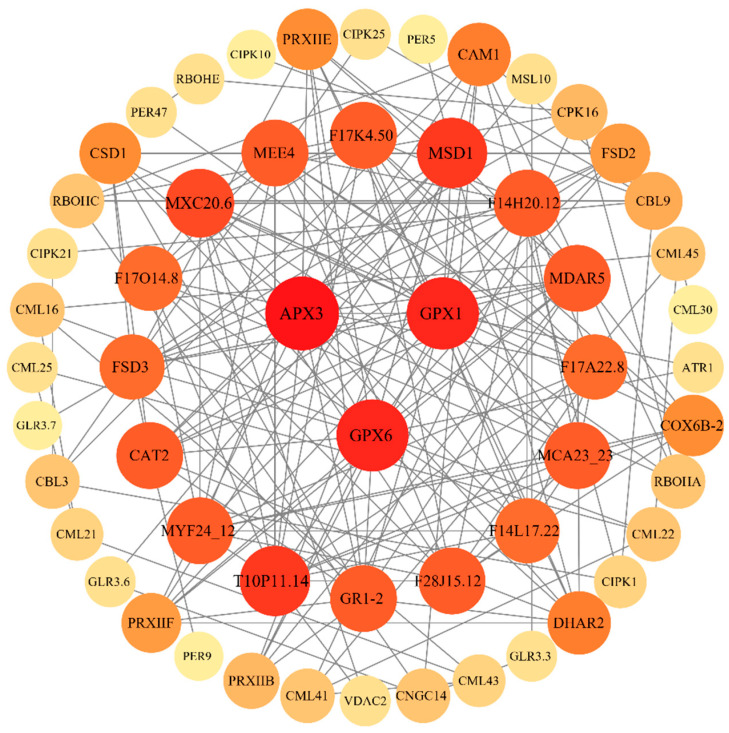
Protein–protein interaction network of DEGs associated with Ca^2+^ signaling and ROS signaling. Nodes represent proteins encoded by DEGs; edges represent protein–protein interactions; dark nodes have high degrees and light nodes have low degrees.

To investigate the possible role of Ca^2+^ and ROS signals activated by exogenous calcium under saline–alkaline stress in castor saline–alkaline tolerance, we conducted a protein interaction network analysis of 129 DEGs associated with Ca^2+^ and ROS signaling. Protein–protein interaction network was created using the STRING database for proteins encoded by these DEGs and displayed with Cytoscape. Under medium confidence settings, nodes with no or minimal interactions with other proteins were excluded. Each node represents a gene (protein), with edges indicating interactions. The more edges a node has, the more central that gene is to the network. *APX3* (LOC8260651), *GPX1* (LOC8274172), and *GPX6* (LOC8266462) were the hub genes in the interaction network (Figure 6). This indicates that crosstalk between Ca^2+^ and ROS signaling takes place, ultimately impacting antioxidant enzymes.

### 2.7. RT-qPCR

**Figure 7:**
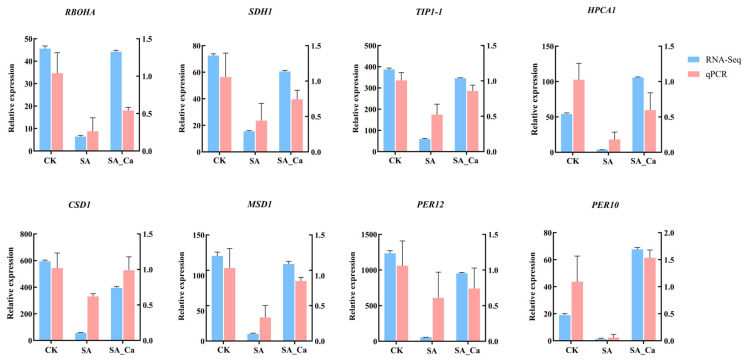
Verification of the expression of DEGs by RT-qPCR. CK: control; SA: saline–alkaline stress; SA_Ca: saline–alkaline stress with calcium treatment.

In this study, we conducted RT-qPCR analysis on eight randomly selected DEGs to confirm the reliability of the RNA-Seq data. Results indicated that the relative expression levels of the eight DEGs obtained by RT-qPCR were highly consistent with the RNA-Seq data. These findings verified the reliability of the differential expression analysis data derived from RNA-Seq (Figure 7).

## 3. Discussion

Ca^2+^ function as signaling molecules that enhance antioxidant capacity and regulate signal transduction pathways in plants. However, the role of calcium in mediating the response of castor seedlings to saline–alkaline conditions remains largely unexplored, and its underlying mechanisms are seldom investigated. To elucidate calcium’s role in the antioxidant response of castor roots under saline–alkaline stress, we analyzed key antioxidant system indicators and gene expression profiles under various treatment conditions. Exogenous calcium pretreatment led to the upregulation of genes coding for antioxidant enzymes, increased antioxidant enzyme activity, reduced oxidative damage, and enhanced castor seedlings’ tolerance to saline–alkaline stress. Furthermore, exogenous calcium treatment was found to upregulate the expression of genes associated with Ca^2+^ and ROS signaling pathways, thereby promoting signal transduction.

MDA levels are used to evaluate the extent of cell membrane damage [32]. Many studies indicate that stress leads to excessive ROS accumulation in plant cells, which triggers membrane lipid peroxidation, and plants adapt to these stresses by enhancing antioxidant enzyme activity [33,34,35]. Ca^2+^ modulates antioxidant enzyme activity and maintains membrane lipid unsaturation, strengthening plant tolerance to stress [12]. In our study, exogenous calcium pretreatment markedly reduced MDA and ROS levels in castor roots subjected to saline–alkaline stress. To examine calcium’s effect on lipid peroxidation and oxidative damage under saline–alkaline stress, we assessed SOD, POD, and CAT activity, discovering that exogenous calcium increases antioxidant enzyme activity in castor roots under stress. Interestingly, in our study, POD and CAT activities increased under saline–alkaline stress, while SOD activity showed the opposite trend, consistent with the findings presented in [36]. Other research has shown that SOD activity decreases in two types of castor leaves under salt stress [37]. The concentration and duration of stress could be the primary reasons for these results [38]. To delve into the regulatory mechanism of exogenous calcium for antioxidant enzyme activity, we analyzed SOD, POD, and CAT-coding genes, discovering that gene expression trends mirrored antioxidant enzyme activity across treatments. This finding aligns with research on the effects of calcium treatment on antioxidant enzymes and related genes in sweet cherries during storage [39]. Under saline–alkaline stress, all SOD-coding genes were downregulated, resulting in reduced SOD activity. Furthermore, the elevated POD activity under saline–alkaline stress may be linked to the upregulation of the *PER46* gene. Interestingly, the expression of the two CAT-coding genes was opposite between the SA and SA_Ca treatments. To explore this, we conducted a domain alignment analysis on the two CAT2 genes. Our analysis revealed that the *CAT2* (LOC8268764) gene possesses one additional CAT active site domain compared to *CAT2* (LOC107261240). Research has shown that structural domains can influence an enzyme’s catalytic rate and variations in enzyme activity with substrates [40]. This indicates that the differing expression of the two CAT2 genes correlates with hydrogen peroxide content. The upregulation of the *CAT2* (LOC8268764) gene under saline–alkaline stress results in heightened CAT activity. On the other hand, the *CAT2* (LOC107261240) gene primarily contributes to a calcium-induced CAT activity increase under saline–alkaline stress. This study is the first to reveal that the CAT2 genes exhibit varied responses to calcium, which are attributed to domain differences. The above results indicate that the application of exogenous calcium under saline–alkaline stress alleviates oxidative damage in castor roots by upregulating antioxidant enzyme-coding gene expression and increasing antioxidant enzyme activity.

The crucial role of Ca^2+^ in many signal transduction pathways is widely recognized [41,42]. Voltage-dependent anion channels (VDAC), mechanosensitive ion channels (MSL), calcium ATPases (ACA), cyclic nucleotide-gated channels (CNGC), and glutamate receptor-like proteins (GLR) are essential for calcium ion transport, Ca^2+^ signaling, and environmental stress tolerance [43,44,45,46,47]. Our research found that all calcium ion channels and calcium signal sensor-related genes, except *CBL9* and *CIPK1*, had their expression suppressed by saline–alkaline stress but were upregulated with exogenous calcium. Melatonin-mediated drought stress responses may activate Ca^2+^ signaling through upregulated expression of CNGC, CaM/CML, and CDPK family genes [48]. Additionally, *OsCPK12* has been proven to phosphorylate *OsCATA* and *OsCATC*, enhancing CAT activity and sustaining H_2_O_2_ homeostasis [49]. Interestingly, the expression of the *CBL9* and *CIPK1* genes was opposite that of the other genes. Earlier research proposed that the *CBL9-CIPK1* module acts as a calcium sensor that negatively regulates drought stress by adjusting the ABA receptor PYL [50]. Thus, we hypothesize that exogenous calcium could enhance castor tolerance to saline–alkaline stress by suppressing *CBL9-CIPK1* transcription, but further research is needed to clarify the mechanism. These findings suggest that exogenous calcium upregulates calcium ion channels and calcium signal sensor-related genes, activating intracellular Ca^2+^ signaling and enhancing castor root tolerance to saline–alkaline stress.

RBOHs are capable of initiating ROS signal generation within the apoplast. ROS in the apoplast can interact directly or indirectly with the ROS receptor HPCA1, leading to the oxidation of various Ca^2+^ channels [51]. Elevated apoplastic H_2_O_2_ promotes Ca^2+^ influx into the cell, leading to more H_2_O_2_ production in the apoplast and further Ca^2+^ influx [52]. Additionally, apoplast-generated ROS must pass through aquaporins (AQPs) to enter the cell, initiating cytoplasmic signaling cascades [53]. Our study revealed that exogenous calcium upregulated all AQP-related genes under saline–alkaline stress. Research has shown that trehalose induces *CmPIP2-3* gene expression, conducting redox signals by facilitating apoplastic H_2_O_2_ transport into the cytoplasm and stimulating downstream antioxidants to boost cold tolerance in melon seedlings [54]. Hence, under saline–alkaline stress, exogenous calcium likely contributes to ROS signaling by upregulating AQP-related gene expression. Our study showed that saline–alkaline stress downregulated all ROS signaling and ROS sensor-related genes while exogenous calcium upregulated them. In a study on cadmium stress tolerance in wheat, the opposite results were observed, with *RBOHA/C/E* gene expression markedly increasing under cadmium stress [55]. Research also indicates that lasers can trigger a transient, RBOH-dependent H_2_O_2_ burst, serving as a downstream signal to mitigate salt stress [56]. The discrepancies in findings might be due to the varied functions of ROS. In non-photosynthetic plant tissues, mitochondria are regarded as the main source of ROS generation [57]. In plants, the mitochondrial electron transport chain (mtETC) comprises four major respiratory complexes: NADH dehydrogenase (Complex I), succinate dehydrogenase (Complex II), cytochrome c reductase (Complex III), and cytochrome c oxidase (Complex IV). Complex II is involved in the defense response to several abiotic stresses [58,59]. Research found that mitochondrial Complex I inhibition decreases ROS accumulation, thereby improving salt tolerance in Arabidopsis [60]. Salicylic acid activates succinate dehydrogenase (SDH) near its ubiquinone binding site, promoting plant stress signaling by enhancing mitochondrial H_2_O_2_ production rates [61]. Our study showed that exogenous calcium application under saline–alkaline stress upregulated Complex I/II-related gene expression in mitochondria. The upregulation of Complex II-related genes under calcium treatment might be because TCA cycle metabolites suppress succinate oxidation [62]. These findings suggest that exogenous calcium under saline–alkaline stress increases the expression of ROS-generating genes, activating ROS signaling pathways in the apoplast and organelles and enhancing the castor’s tolerance to saline–alkaline stress.

Research has shown that HPCA1 connects H_2_O_2_ accumulation in the apoplast with Ca^2+^ signaling and RBOH-mediated ROS generation, thus facilitating plant stress tolerance [51]. The apoplast–cytoplasm interface is emerging as a primary hub for ROS-related signaling transduction during various stress responses [23]. Furthermore, the cytoplasm hosts multiple signaling hubs, such as calcium-dependent protein kinases (CDPK/CPK), calcineurin B-like interacting protein kinases (CIPK), Rho of plants (ROP), and various phosphatases (PP2A and PP2C), which integrate ROS and Ca^2+^ signals [63]. In our study, exogenous calcium upregulated HPCA1 and signaling hub-related genes under saline–alkaline stress. Antioxidant enzymes can serve as nodes in Ca^2+^–ROS crosstalk. For example, a Ca^2+^-dependent peroxidase known as Euphorbia peroxidase (ELP) is activated by calmodulin (CaM) [64]. In addition, protein interaction network analysis indicates that exogenous calcium facilitates Ca^2+^–ROS signaling crosstalk, which may affect antioxidant enzymes and contribute to castor’s saline–alkaline tolerance. To clearly depict the Ca^2+^–ROS signaling crosstalk under saline–alkaline stress, we proposed a model illustrated in Figure 5A. Further research is needed to understand the relationship between Ca^2+^–ROS signaling crosstalk and antioxidant enzyme activity under saline–alkaline stress.

## 4. Materials and Methods

### 4.1. Plant Material

Castor bean seeds (Zibi 5) were obtained from the Shandong Academy of Agricultural Sciences, China. The seeds were soaked in a water bath at 40 °C for 10 h, air-dried, and sown in 18-cm diameter plastic pots filled with sterilized nutrient soil (charcoal soil:vermiculite = 3:1). Plants were grown under controlled conditions in an incubator (14-h light/10-h dark photoperiod, 26/20 °C day/night temperature, 60% relative humidity) for 7 days with regular watering. Subsequently, the seedlings were transferred to hydroponic pots containing 1/4 Hoagland nutrient solution for 7 days, with the solution refreshed every two days. Uniformly grown seedlings with fully expanded cotyledons were selected for experiments.

### 4.2. Experimental Design

The experiment comprised three treatment groups: (1) distilled water for 50 h (CK), (2) 50 mmol/L NaHCO_3_ for 50 h (SA), and (3) 15 mmol/L CaCl_2_ pretreatment for 24 h—followed by washing—and then 50 mmol/L NaHCO_3_ for 36 h (SA_Ca). The NaHCO_3_ concentration was optimized based on preliminary experiments to induce significant stress without hindering measurable indicators, while the CaCl_2_ concentration was set at 15 mmol/L [65]. Each treatment included three biological replicates in a randomized block design, comprising a total of nine pots, each containing six seedlings. In the SA_Ca group, roots were pretreated with 15 mmol/L CaCl_2_ under dark conditions for 24 h, conducted separately to ensure no interference with subsequent NaHCO_3_ treatments. Roots from the CK and SA groups were soaked in distilled water under identical conditions. After pretreatment, the castor bean roots were thoroughly rinsed with deionized water, and plants (except CK) were exposed to the respective treatments for 36 h, during which physiological indicator differences were most pronounced. The roots were then harvested, immediately frozen in liquid nitrogen, and stored at −80 °C for subsequent physiological analyses and transcriptomic sequencing.

### 4.3. Physiological Index of Plants

Malondialdehyde (MDA) content was quantified based on the level of thiobarbituric acid reactive substances (TBARSs) in root samples, expressed as nmol/g fresh weight, using an extinction coefficient of 155 mM^−1^cm^−1^ [66]. Absorbance was measured at 532, 600, and 450 nm [67]. Superoxide anion (O_2_^.−^) content was determined using the hydroxylamine hydrochloride oxidation method, with absorbance recorded at 530 nm [68]. The resulting yellow precipitate was dissolved in 1 mmol/L sulfuric acid, and its absorbance was measured at 410 nm.

Root enzyme extraction was performed using 0.5 g of tissue homogenized in 50 mmol/L phosphate buffer (pH 7.8). After centrifugation (4 °C, 13,926× *g*), the supernatant was used for measuring SOD, POD, and CAT activities. SOD activity was determined using the nitroblue tetrazolium (NBT) photoreduction method [69]; samples were exposed to uniform light for 30 min, and absorbance was measured at 560 nm using a UV spectrophotometer (calibrated to zero after 30 min of dark incubation). One unit (U) of SOD activity was defined as the amount of enzyme required to inhibit NBT reduction by 50% in fresh samples. POD and CAT activities were measured following the method of Cakmak and Marschner [70]. CAT activity was determined by monitoring H_2_O_2_ absorbance decline at 240 nm and converting it into enzymatic units (U), defined as the amount of enzyme needed to decompose 1 μmol of H_2_O_2_ per minute. POD activity was measured by monitoring absorbance changes at 470 nm every 15 s due to its reaction with guaiacol. Protein concentration was quantified using the Bradford method [71].

### 4.4. RNA Isolation, Library Construction and RNA-Seq

The RNA from the total samples was extracted and cleansed using TRIzol (thermofisher, 15596018, Waltham, MA, USA) following the manufacturer’s supplied methodology. The quantity and purity of the total RNA were subsequently assessed using a NanoDrop ND-1000 instrument (NanoDrop, Wilmington, DE, USA), while the integrity of the RNA was evaluated using a Bioanalyzer 2100 device (Agilent, CA, USA). Concentrations > 50 ng/μL, RIN values > 7.0, and total RNA > 1 μg were sufficient for downstream experiments. The mRNA containing PolyA (polyadenylate) was selectively isolated using oligo(dT) magnetic beads. The mRNA that was obtained was broken down into smaller fragments, and complementary DNA (cDNA) was then created. The cDNA was corrected at the ends, and the suitable fragments were chosen for bridging PCR amplification to create a library with a fragment size of 300 bp ± 50 bp (specific to one strand). Ultimately, the process of double-end sequencing was carried out utilizing the Illumina Novaseq 6000 machine at Shanghai Biotree Biotech Co., Ltd. (Shanghai, China).

### 4.5. Real-Time Quantitative Polymerase Chain Reaction (RT-qPCR) Analysis

Quantitative PCR (qPCR) was used to validate the relative expression levels of eight differentially expressed genes (DEGs). Total RNA was extracted using the RNAprep Pure Plant Kit (Tiangen, Beijing, China) according to the manufacturer’s protocol. Castor bean samples were subjected to RNA extraction and purification, followed by conversion to complementary DNA (cDNA) using the TransScrip Reverse Transcription Kit from TransGen Biotech. The qPCR reaction was conducted using a 16 μL combination consisting of 8 μL of 2× M5 HiPer SYBR Premix Es Taq, 2 μL of cDNA template, 0.3 μL of each primer (10 μM), and 5.36 μL of ddH_2_O. The PCR amplification conditions consisted of an initial denaturation step at 95 °C for 10 min, followed by 40 cycles of denaturation at 95 °C for 15 s and annealing at 60 °C for 30 s. The expression levels of eight DEGs identified from RNA-Seq were validated using RT-qPCR, with *ACTIN2* as the internal reference gene [72]. The design of specific primers for RT-qPCR analysis was carried out using Primer 5.0 software. The complete list of gene primers may be seen in Table S4.

### 4.6. Statistical Analysis

Data were presented as mean ± standard deviation (SD) from three biological replicates. A one-way analysis of variance (ANOVA) was conducted, followed by Tukey’s multiple comparisons test, using GraphPad Prism version 10.0.0 for Windows (GraphPad Software, Boston, MA, USA, www.graphpad.com, accessed on 6 August 2024). The unprocessed sequencing reads were filtered using FASTP (version 0.23.0) and then aligned to the castor genome using HSAT2 with the default settings. The analysis of differentially expressed genes was performed using DESeq2 on Count values, with a threshold condition of |log2FC| > 2 and *p* < 0.05. The process of gene functional annotation and pathway analysis was conducted using the Gene Ontology (GO) and Kyoto Encyclopedia of Genes and Genomes (KEGG). Venn and heat maps were generated using the online website OmicStudio (https://www.omicstudio.cn/tool, accessed on 27 September 2024) and Tbtools (version v1.098) [73]. ClustalX was used to perform multiple sequence comparisons, and the results were displayed using ESPript 3.0 (https://espript.ibcp.fr, accessed on 25 September 2024). The structural domains of genes were examined using InterPro (https://www.ebi.ac.uk/interpro/, accessed on 25 September 2024) [74]. The protein–protein interaction (PPI) networks of proteins encoded by differentially expressed genes (DEGs) were created using the STRING online database (https://cn.STRING-db.org/, accessed on 16 September 2024). In addition, the key genes underwent further screening using Cytoscape 3.10.2. Proteins are represented as nodes, and the crucial interactions between proteins in the PPI network are illustrated by connecting edges. Nodes symbolize proteins, whereas the connecting edges denote the pertinent interactions among proteins within the PPI network. The degree is employed to evaluate the ranking of network nodes [75].

## 5. Conclusions

Using physiological and transcriptomic analyses, this research explored how exogenous calcium regulates the antioxidant system and activates Ca^2+^–ROS signaling crosstalk, thereby improving castor seedlings’ tolerance to saline–alkaline stress. The results show that exogenous calcium predominantly controls *CAT2* (LOC107261240) and *APX3* (LOC8260651) gene expression, increases POD and CAT activity, removes excess ROS, and mitigates oxidative stress in the roots. Furthermore, exogenous calcium activates endogenous Ca^2+^ and ROS signaling, and the crosstalk between them plays a role in enhancing castor’s tolerance to saline–alkaline stress.

## Data Availability

Data is contained within the article or Supplementary Materials.

## Supplementary Materials

The following supporting information can be downloaded at: https://www.mdpi.com/article/10.3390/ijms252312717/s1.
